# Determining the optimal hematoma volume-based thresholds for surgical and medical strategies in basal ganglia hemorrhage

**DOI:** 10.1007/s10143-025-03403-6

**Published:** 2025-02-20

**Authors:** Chonnawee Chaisawasthomrong, Atthaporn Boongird

**Affiliations:** 1 Division of Neurosurgery, Department of Surgery, Ratchaburi Hospital, Ratchaburi, Thailand; 2 Division of Neurosurgery, Department of Surgery, Faculty of Medicine Ramathibodi Hospital, Mahidol University, Bangkok, 10400 Thailand

**Keywords:** Basal ganglia hemorrhage, Hematoma volume, Medical treatment, Surgical intervention

## Abstract

Hematoma volume is a significant concern in basal ganglia hemorrhage, with no clear cutoff to guide the choice between conservative and surgical management, particularly for larger hematomas where the optimal approach remains controversial. This study aimed to determine the maximum hematoma volume suitable for conservative treatment and the volume that necessitates surgical intervention in patients with basal ganglia hemorrhage. A total of 387 cases of basal ganglia hemorrhage from 2019 to 2021 were analyzed, evaluating patient demographics, medical history, and initial CT brain scans to assess hematoma volume. Outcomes of medical and surgical treatments were compared using multivariate logistic and Cox regression analysis. For patients treated with medical management alone, mortality rates did not differ significantly between hematoma volumes of 10–39.9 mL and those under 10 mL. Receiver operating characteristic (ROC) curve analysis identified a cutoff volume of 45.3 mL, with a sensitivity of 80.82% and specificity of 91.67% for predicting survival. Kaplan–Meier survival analysis revealed a reduced mortality hazard ratio (0.17) with surgical intervention for hematomas exceeding 45.3 mL. However, surgical treatment for volumes under 30 mL was associated with higher mortality compared to medical management. Surgical intervention showed a clear survival benefit for hematoma volumes of at least 60 mL, while conservative treatment remained appropriate for volumes up to 45.3 mL. For volumes between 45.3 mL and 59.9 mL, the decision to operate should be guided by the surgeon’s judgment and patient-specific factors such as comorbidities, brain atrophy. In conclusion, conservative management is effective for hematomas up to 45.3 mL, while surgical intervention is absolutely indicated for volumes of 60 mL or more. These findings provide valuable guidance for optimizing treatment strategies in basal ganglia hemorrhage.

## Introduction

Spontaneous intracerebral hemorrhage (SICH) is a common occurrence, with an annual incidence ranging between 11 and 23 cases per 100,000 [1]. In the United States, there were 107,590 identified discharges related to ICH, and a craniotomy procedure code was detected in 7,518 instances (7.0%) [2]. The mortality rate for ICH within 30 days is 40–50%, which is double that of ischemic stroke. Only 27% of patients achieve functional independence after 90 days [3, 4]. Following the evacuation of intracranial hemorrhage, the 30-day mortality rate was 23.3% [5]. Notably, mortality was higher among individuals aged 65 years and older (*p* = 0.020), deep-seated location and intraventricular hemorrhage [6]. The indication for surgical intervention for SICH remains controversial [7]. Some studies suggest that surgery is warranted when the hematoma volume exceeds 30 ml [8]. In cases of secondary neurological deterioration with a hematoma volume ranging from 50 to 60 ml, performing an open craniotomy and evacuation the hematoma can be life-saving [9]. In clinical practice, there were numerous patients presenting to the emergency room with more than 30 ml of SICH, yet their neurological examination was intact, and they could survive without further surgical intervention. The role of open craniotomy for early hematoma drainage after intracranial hemorrhage remains a topic of hot debate, and randomized controlled trials have failed to demonstrate its benefit in terms of mortality or functional outcomes [10]. The STICH II results confirm that early surgery does not increase the rate of death or disability at 6 months and might offer a small but clinically relevant survival advantage for patients with spontaneous superficial intracerebral hemorrhage without intraventricular hemorrhage [11, 12].

This paper aimed to conduct a retrospective comparison between medical treatment and surgical intervention in basal ganglia hemorrhage across different volumes. The objective is to identify the maximum volume suitable for conservative treatment and the volume that serves as an absolute indication for surgery.

## Materials and methods

A retrospective data collection was performed on patients diagnosed with basal ganglia hemorrhage at Ratchaburi Hospital in Ratchaburi Province, Thailand, between the years 2019 and 2021. The patients were closely monitored for a period of 30 days following the occurrence of intracerebral hemorrhage during their hospital stay. Any incomplete medical records, patients who demonstrate resistance towards seeking hospital care or exhibit attempts to evade medical treatment, instances of intracerebral hemorrhage attributed to secondary causes such as a bleeding tumor, ruptured aneurysms, arteriovenous malformation, arteriovenous fistula, hemorrhagic transformation, venous sinus thrombosis, coagulopathy and patients who experienced hematoma expansion during treatment, were excluded from the research analysis. History and diagnostic imaging results were gathered from all medical records. Personal history and general information, such as gender, age, underlying diseases, alcohol consumption, and smoking habits, were also recorded. Additionally, The Radiographic imaging history included volume of hematoma, interpreted by the radiologists using Synapse software version 4.4.210 (Fujifilm Thailand, Bangkok, Thailand).

**Fig. 1:**
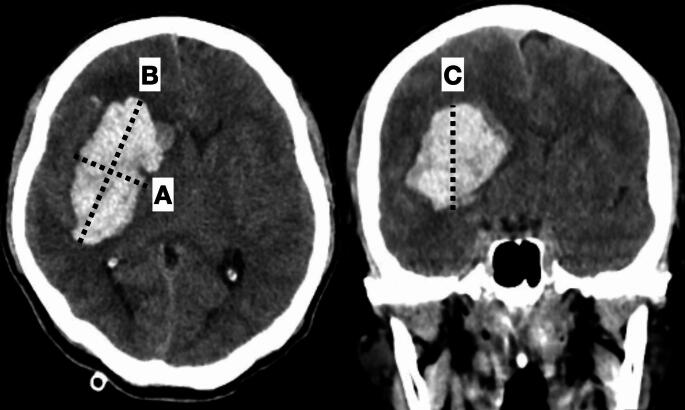
Volume of the hemorrhage **A** x **B** x **C**/2. **A** dash line, the greatest hemorrhage diameter on the CT axial view; **B** dash line, the diameter perpendicular to **A** dash line; **C** dash line, the maximal diameter of the hemorrhage on the coronal view

The volume of the hematoma within the basal ganglia was calculated without including intraventricular hemorrhage volume, using the formula A × B × C / 2. Here, A represents the greatest hemorrhage diameter on the CT axial view, B represents the diameter perpendicular to A, and C represents the maximal diameter of the hematoma on the coronal view (Fig. 1). The data analysis was performed using Stata software version 17 (StataCorp, College Station, TX, USA).

The treatment strategy reserves hyperosmotic agents for patients with clinical signs of increased intracranial pressure, while surgical options, including ventriculostomy for obstructive hydrocephalus, mini-craniectomy (a craniotomy flap averaging 3 cm in diameter), decompressive craniectomy (a larger flap typically averaging 12 cm in diameter), and conventional craniotomy with clot removal, are determined by the degree of brain edema and the individual surgeon’s preference.

An analysis was undertaken to determine the maximum volume that could be managed through medical treatment by comparing outcomes among patients who either survived or died while receiving medical treatment across various hematoma volumes. Simultaneously, the study aimed to identify the minimum volume that could be effectively treated with surgical intervention, minimizing morbidity and mortality. This comparison involved assessing the mortality outcomes of patients who received medical treatment against those who underwent surgical intervention at different hematoma volumes. The goal was to establish the volumetric threshold for medical treatment associated with survival, and the minimum volume necessitating surgical intervention while minimizing adverse outcomes, focusing solely on the volume of hematoma without considering clinical manifestations of consciousness or signs of herniation. For quantitative data, the Student’s t-test or Mann-Whitney U test was used, while categorical data were analyzed with the chi-squared test. Univariate and multivariate logistic regression analyses were applied to account for confounding variables, and Cox regression analysis was used for survival analysis at the cutoff volume. A significance level of *P* < 0.05 was considered statistically significant.

## Results

In Ratchaburi Hospital from 2019 to 2021, a total of 387 cases of basal ganglia hemorrhage were identified, significant differences were observed in the median hematoma volume between the surgical and medical treatment groups (58 mL [IQR: 36.2–80] vs. 22 mL [IQR: 9–55], *p* < 0.001). Other variables, including age, sex, coronary artery disease (CAD), chronic kidney disease (CKD), smoking, and alcohol consumption, showed no statistically significant differences between the groups (Table 1).

**Table 1:** Demographic data for patients with basal ganglia hemorrhage

Variables	Total (*N* = 387)	Surgery (*n* = 132)	Medical (*n* = 255)	OR	95%CI	*p*-value
**Age Mean ± SD**	58.04 ± 13.01	56.86 ± 12.26	58.65 ± 13.36	0.99	0.97-1.00	0.200
**Male**,** n (%)**	274(70.80)	91(33.21)	183(66.79)	0.87	0.55–1.38	0.562
**Female**,** n (%)**	113(29.20)	41(36.28)	72(64.72)			
**CAD**,** n (%)**	17(4.39)	4(3.03)	13(5.10)	0.58	0.19–1.82	0.352
**CKD**,** n (%)**	23(5.94)	4(3.03)	19(7.45)	0.39	0.13–1.17	0.092
**Smoking**,** n (%)**	130(33.59)	44(33.33)	86(33.73)	0.98	0.52–1.51	0.654
**Alcoholic**,** n (%)**	175(45.22)	61(46.21)	114(44.71)	1.06	0.70–1.62	0.778
**Volume of hematoma Median(IQR)**	34.2(14.35,69.85)	58(36.2,80)	22(9,55)	1.01	1.01–1.02	0.000*

SD, standard deviation; IQR, Interquartile range

OR, odds ratio

* P-value < 0.05

Among 255 patients with basal ganglia hemorrhage who received medical treatment only, significant differences were observed between survivors and non-survivors. Non-survivors had higher hematoma volumes (median 79.8 mL [IQR: 55–117.25] vs. 16.1 mL [IQR: 6.4–27.4], *p* < 0.001) and lower Glasgow Coma Scale (GCS) scores (median 3 [IQR: 2–5] vs. 14 [IQR: 11–15], *p* < 0.001). Survivors were more likely to have reactive pupils (98.9% vs. 23.29%, *p* < 0.001). CKD and CAD were significantly more prevalent in non-survivors, with CKD (*p* = 0.014) and CAD (*p* < 0.001) as potential predictors of poor outcomes. Tranexamic acid use was associated with improved survival (*p* = 0.037). CKD, CAD, and tranexamic acid were identified as potential confounding factors and should be further analyzed using multivariate analysis to exclude their confounding effects (Table 2).

**Table 2:** Demographic data of patients undergoing medical treatment for basal ganglia hemorrhage

Variables	Total (*N* = 255)	Survived (*n* = 182)	Died (*n* = 73)	OR	95%CI	*p*-value
**Age Mean ± SD**	58.65 ± 13.36	56.92 ± 12.05	62.96 ± 15.42	0.97	0.94–1.02	0.281
**Male**,** n (%)**	183(71.76)	137(74.86)	46(25.14)	1.79	0.57–4.98	0.349
**Female**,** n (%)**	72(28.24)	45(62.50)	27(37.50)			
**Underlying disease**						
**CAD**,** n (%)**	13(5.10)	2(1.1)	11(15.07)	0.06	0.00-0.10	0.000*
**CKD**,** n (%)**	19(7.45)	8(4.4)	11(15.07)	0.26	0.04–0.70	0.014*
**Smoking**,** n (%)**	86(33.73)	63(34.62)	23(31.51)	1.15	0.30–2.07	0.628
**Alcoholic**,** n (%)**	114(44.71)	84(46.15)	30(41.1)	1.23	0.38–2.48	0.942
**Initial Examination**						
**Glasgow coma scale**,** median (IQR)**	12(5,15)	14(11,15)	3 (2,5)	1.74	1.54–1.97	0.000*
**Reactive Pupils**,** n (%)**	197(77.25)	180 (98.90)	17 (23.29)	296.47	66.45-1322.77	0.000*
**Volume of hematoma Median(IQR)**	21.2(9,55)	16.1(6.4,27.4)	79.8(55,117.25)	0.95	0.93–0.96	0.000*
**Medication**						
**Mannitol**,** n (%)**	44 (17.25)	28(15.38)	16(21.92)	0.65	0.33–1.29	0.214
**Glycerol**,** n (%)**	24 (9.41)	14(7.69)	10(13.70)	0.53	0.22–1.24	0.143
**Vitamin K**,** n (%)**	43(16.86)	34(18.68)	9(12.33)	1.63	0.74–3.60	0.224
**Tranexamic acid**,** n (%)**	45(17.65)	38(20.88)	7(9.59)	0.49	1.05–5.86	0.037*

SD, standard deviation; IQR, Interquartile range

* P-value < 0.05

In a study of 132 patients with basal ganglia hemorrhage undergoing surgical intervention, key differences were observed between surviving and deceased patients. Surviving patients exhibited higher GCS scores (median 7 [IQR: 6– 10] vs. 5 [IQR: 4–6], *p* = 0.001) and a higher rate of reactive pupils (90.91% vs. 54.55%, *p* < 0.001). Deceased patients were more likely to undergo ventriculostomy, which was associated with worse outcomes (27.27% vs. 9.09%, *p* = 0.012). Mini-craniectomy, decompressive craniectomy, or craniotomy with clot removal showed no statistically significant difference between deceased and surviving patients. Additionally, although deceased patients had a trend toward higher hematoma volumes (median 71.8 mL [IQR: 41.5–92.7] vs. 54.5 mL [IQR: 33.5–74.4]), this difference did not reach statistical significance (*p* = 0.161) (Table 3).

**Table 3:** Demographic data of patients undergoing surgical intervention for basal ganglia hemorrhage

Variables	Total (*N* = 132)	Survived (*n* = 99)	Died (*n* = 33)	OR	95%CI	*p*-value
**Age Mean ± SD**	56.86 ± 12.26	57.41 ± 11.62	55.21 ± 14.08	1.01	0.98–1.05	0.371
**Male**,** n (%)**	91(68.94)	68(68.69)	23(69.70)	0.95	0.41–2.24	0.914
**Female**,** n (%)**	41(31.06)	31(31.31)	10(30.30)			
**Underlying disease**						
**CAD**,** n (%)**	4(3.03)	2(2.02)	2(6.06)	0.32	0.04–2.36	0.264
**CKD**,** n (%)**	4(3.03)	2(2.02)	2(6.06)	0.32	0.04–2.36	0.264
**Smoking**,** n (%)**	44(33.33)	31(31.31)	13(39.39)	0.70	0.31–1.59	0.395
**Alcoholic**,** n (%)**	61(46.21)	49(49.49)	12(36.36)	1.72	0.76–3.86	0.193
**Examination**						
**Glasgow coma scale**,** median (IQR)**	6.5(5,10)	7(6,10)	5(4,6)	1.30	1.12–1.53	0.001*
**Reactive Pupils**,** n (%)**	108(81.82)	90(90.91)	18(54.55)	8.33	3.16–21.96	0.000*
**Volume of hematoma Median(IQR)**	58(36.2,74.4)	54.5(33.5,74.4)	71.8(41.5,92.7)	0.99	0.98-1.00	0.161
**Medication**						
**Mannitol**,** n (%)**	55(41.67)	38(38.38)	17(51.52)	0.58	0.27–1.30	0.187
**Glycerol**,** n (%)**	11(8.33)	6(6.06)	5 (15.15)	0.36	0.10–1.27	0.112
**Vitamin K**,** n (%)**	49(37.12)	39(39.39)	10(30.30)	1.50	0.64–3.48	0.351
**Tranexamic acid**,** n (%)**	58(43.94)	45(45.45)	13(39.39)	1.28	0.57–2.86	0.544
**Surgical intervention**						
**Ventriculostomy**,** n (%)**	18(13.64)	9(9.09)	9(27.27)	0.27	0.10–0.75	0.012*
**Craniotomy with clot removal**,** n (%)**	17(12.88)	14(14.14)	3(9.09)	1.65	0.44–6.13	0.457
**Mini-Craniectomy with clot removal**,** n (%)**	92(69.70)	72(72.73)	20(60.61)	1.73	0.76–3.96	0.192
**Decompressive Craniectomy with clot removal**,** n (%)**	13(9.85)	9(9.09)	4(12.12)	0.72	0.21–2.53	0.614

* P-value < 0.05

Focusing on the difference in hematoma volume among patients undergoing medical treatment only, the analysis revealed distinct outcomes based on various volume ranges. Mortality rates varied significantly across different hematoma volume ranges. The analysis showed that smaller hematoma volumes (0–9.9 mL) were associated with very low mortality (5.97% of deaths). However, as hematoma volume increased, the mortality rate rose notably. Hematomas of 40–49.9 mL had a mortality rate of 33.33% (*p* = 0.003), while volumes of 50–59.9 mL showed a mortality rate of 60% (*p* < 0.001). Even higher mortality rates were observed in patients with hematomas between 60 and 69.9 mL (80%, *p* < 0.001), 70–79.9 mL (77.78%, *p* < 0.001), and ≥ 80 mL (87.5%, *p* < 0.001). These findings indicate a strong association between increasing hematoma volume and higher mortality risk in basal ganglia hemorrhage patients treated medically (Table 4).

**Table 4:** Analysis of varying hematoma volumes in patients undergoing medical treatment for basal ganglia hemorrhage

Imaging				Died		
Variables	Total (*N* = 255)	Died (*n* = 73)	Survived (*n* = 182)	aOR**	95%CI	*p*-value
**Volume(ml),** *n* **(%)**						
**0-9.9**	**67(26.27)**	4(5.97)	63(94.03)	-	-	-
**10-29.9**	**89(34.90)**	8(8.99)	81(91.01)	3.47	0.63–19.20	0.154
**30-39.9**	**18(7.06)**	1(5.56)	17(94.44)	4.01	0.30-54.38	0.296
**40-49.9**	**12(4.71)**	4(33.33)	8(66.67)	23.74	2.85-197.73	0.003*
**50-59.9**	**10(3.92)**	6(60.00)	4(40.00)	68.39	8.19-571.29	0.000*
**60-69.9**	**10(3.92)**	8(80.00)	2(20.00)	215.52	22.53-2061.88	0.000*
**70-79.9**	**9(3.53)**	7(77.78)	2(22.22)	232.22	23.67-2277.99	0.000*
**≥ 80**	**40(15.69)**	35(87.5)	5(12.5)	370.93	55.78-2466.53	0.000*

aOR, adjusted odds ratio

* P-value < 0.05

** adjusted with CAD, CKD, Tranexamic acid

The comparison of survival rates between surgical management and medical treatment revealed significant findings across different hematoma volume groups. Notable differences in survival rates were observed. For patients with smaller hematoma volumes (0–29.9 mL), medical treatment showed superior survival rates, with 92.21% surviving versus 76.19% for surgery. In contrast, surgical intervention demonstrated a clear survival benefit for patients with larger hematoma volumes. For volumes of 60–69.9 mL, surgery had a survival rate of 87.5% compared to 20% for medical treatment. Similarly, for volumes of 70–79.9 mL, surgical intervention resulted in a survival rate of 71.43% compared to 22.22% for medical treatment. Finally, for hematoma volumes ≥ 80 mL, surgical treatment maintained a survival advantage, with 61.76% surviving versus 16.67% for medical treatment (Table 5).

**Table 5:** Comparison of the outcomes of surgical intervention versus medical treatment in individuals with diverse volumes of basal ganglia hemorrhage

Imaging		Surgery(*n* = 132)		Medical(*n* = 255)		Survived		
Variables	Total (*N* = 387)	Survived (*n* = 99)	Died (*n* = 33)	Survived (*n* = 182)	Died (*n* = 73)	aOR**	95%CI	*p*-value
**Volume(ml),** *n* **(%)**								
**0-29.9**	**175(45.22)**	16(76.19)	5(23.81)	142(92.21)	12(7.79)	0.12	0.03–0.50	0.003*
**30-39.9**	**34(8.79)**	13(81.25)	3(18.75)	17(94.44)	1(5.56)	0.21	0.02–2.39	0.209
**40-49.9**	**31(8.01)**	16(84.21)	3(15.79)	8(66.67)	4(33.33)	1.71	0.22–13.41	0.608
**50-59.9**	**22(5.68)**	9(75)	3(25)	4(40)	6(60)	4.45	0.55–36.26	0.163
**60-69.9**	**26(6.72)**	14(87.5)	2(12.5)	2(20)	8(80)	24.50	1.79-336.23	0.017*
**70-79.9**	**23(5.94)**	10(71.43)	4(28.57)	2(22.22)	7(77.78)	8.75	1.24–61.68	0.029*
**>=80**	**76(19.64)**	21(61.76)	13(38.24)	7(16.67)	35(83.33)	5.61	1.80-17.45	0.003*

aOR, adjusted odds ratio

*P-value < 0.05

** adjusted with CAD, CKD, Tranexamic acid

Post-treatment outcomes in surviving basal ganglia hemorrhage patients reveal significant differences between surgical intervention and medical treatment. Surgical intervention is associated with a significantly longer median length of hospital stays (LOS) (16 days vs. 7 days, *p* = 0.002). Pneumonia occurred significantly more frequently in the surgical group (44.44%) compared to the medical group (14.75%). Pre-treatment and post-treatment mRS scores indicate better outcomes in the medical group. The overall change in mRS scores is minimal in both groups, though the medical group shows a slight advantage, especially right side hemorrhage. (Table 6)

**Table 6:** Comparison of post-treatment outcomes between surgical intervention and medical treatment in survived basal ganglia hemorrhage patients

Variables	Total (*N* = 281)	Surgery(*n* = 99)	Medical(*n* = 182)	uOR	aOR**	95%CI	*p*-valve
Hospital stay , days (IQR)	9(4,20)	16(8,25)	7(3,15)	1.03	1.02	1.01–1.04	0.002
**Complications**							
Pneumonia, n (%)	71(25.18)	44(44.44)	27(14.75)	4.62	4.35	2.41–7.85	0.000*
Urinary tract infection, n (%)	22(7.83)	6(6.12)	16(8.74)	0.68	0.61	0.22–1.68	0.341
Septicemia, n (%)	8(2.84)	5(5.05)	3(1.64)	3.19	3.01	0.63–14.36	0.167
Cerebral Infarction, n (%)	2(0.71)	0(0.00)	2(1.09)	N/A	N/A	N/A	N/A
Post operative bleeding, n (%)	3(3.03)	3(3.03)					
Post operative Brain edema, n (%)	4(4.04)	4(4.04)					
**Modified Rankin scale**							
Pre treatment, median (IQR)	5(5,5)	5(5,5)	5(3,5)	3.28	3.10	1.78–5.38	0.000*
Rt side	5(4.5,5)	5(5,5)	5(3,5)	2.52	2.57	1.35–4.90	0.004*
Lt side	5(5,5)	5(5,5)	5(3,5)	7.23	6.67	1.30-34.12	0.023*
Post treatment, median (IQR)	5(3,5)	5(5,5)	4(3,5)	2.84	2.81	1.94–4.09	0.000*
Rt side	5(3,5)	5(5,5)	4(3,5)	2.47	2.63	1.62–4.26	0.000*
Lt side	5(3,5)	5(5,5)	5(3,5)	3.65	3.53	1.78–6.70	0.000*
Change, median (IQR)	0(0,0)	0(0,0)	0(0,1)	0.44	0.45	0.28–0.72	0.001*
Rt side	0(0,0)	0(0,0)	0(0,1)	0.43	0.42	0.21–0.84	0.015*
Lt side	0(0,0)	0(0,0)	0(0,1)	0.45	0.47	0.24–0.93	0.029*

IQR, Interquartile range

uOR, unadjusted odds ratio; aOR, adjusted odds ratio

* P-value < 0.05

** adjusted with CAD, CKD, Tranexamic acid

## Discussion

The medical approach to SICH centers around prompt stabilization and addressing the acute condition of intracranial hemorrhage by identifying and treating the root causes of decreased alertness. This encompasses actively rectifying elevated blood pressure and reversing any coagulopathy [13, 14]. In situations where patients display significant or advancing elevation in intracranial pressure (ICP), or if milder symptoms persist despite initial interventions, osmotic therapy is utilized as a therapeutic measure [15]. In this study, no demographic differences were observed between patients undergoing surgical intervention and those receiving medical treatment. An analysis of patients receiving medical treatment exclusively revealed that tranexamic acid significantly reduced the risk of mortality, while CKD, CAD, and CT brain hematoma volume were significantly associated with an increased mortality rate in cases of basal ganglia hemorrhage. Tranexamic acid has been shown to improve survival in non-surgical patients with basal ganglia hemorrhage by limiting hematoma expansion, as supported by evidence from meta-analyses [16] and randomized controlled trials [17]. Early administration of tranexamic acid (within 2 hours of onset) significantly reduces hematoma growth, thereby improving outcomes in intracerebral hemorrhage. Its antifibrinolytic properties stabilize clots and prevent further bleeding, making it particularly beneficial for medically managed patients. However, tranexamic acid’s usefulness is less pronounced in post-surgical settings. Current evidence highlights its greatest effectiveness in hyperacute medical management [17].

The primary surgical indication reported in studies is for patients in a comatose state (with a GCS score < 8) or those with hematomas exceeding 30 mL [18], or individuals whose intracranial pressure did not normalize with medical management [19, 20]. Some studies suggest avoiding surgery for minor basal ganglia hematomas (25–40 ml), as open craniotomy may worsen long-term outcomes compared to conservative treatment [21], even though a 30 ml volume has traditionally indicated surgery. In real-world situations, many patients exhibit tolerance and maintain autoregulation, enabling their survival. The preceding study identified the factors contributing to 30-day in-hospital mortality. It did not include the volume of hematoma but highlighted the association with the degree of compression of the basal cistern and physical examination [22]. Nevertheless, there is no specific volume that can be exclusively managed through medical treatment. In the medical treatment alone, a group across different hematoma volumes in this study revealed that the 10-39.9 ml group had a non-significantly different likelihood of mortality compared to the less than 10 ml group. It can be concluded that the volume 30-39.9 ml group was not absolutely indicated for surgical intervention and some patients can be tolerated with ICP autoregulation. However, These findings suggest a clear association between increasing hematoma volume, particularly volumes exceeding 40 ml, and higher mortality rates among patients receiving medical treatment compared to the less than 10 ml group.

The summary suggests that for patients with hemorrhage volumes ranging from 0 to 39.9 ml, treatment solely with medication remains a viable option, with a higher likelihood of survival compared to undergoing surgery. This contradicts previous research findings, which focused on patients with SICH with volumes greater than 30 ml [8, 18].

**Fig. 2:**
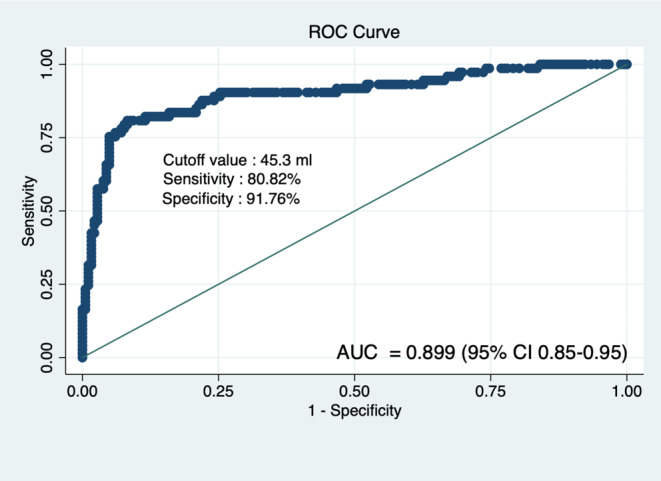
Receiver Operating Characteristic (ROC) curve of hematoma volume in patients undergoing only medical treatment for predicting mortality. AUC mean area under curve; 95% CI, 95% confidence interval

Hematoma volume was a crucial factor considered in constructing the Receiver Operating Characteristics (ROC) curve. The primary objective of the ROC curve was to distinguish between patients who survived and those who succumbed to basal ganglia hemorrhage despite undergoing medical treatment. The area under the curve was 0.899 (95% CI 0.85–0.95), and a cutoff value of 45.3 ml was established. This cutoff value effectively predicted survival with medical treatment alone, achieving a sensitivity of 80.82% (95%CI 69.9–89.1), specificity of 91.76% (95%CI 86.8–95.3), Positive likelihood ratio 9.81 (95%CI 5.96–16.13), Negative likelihood ratio 0.21(95%CI 0.13–0.34), Positive predictive value(PPV) 79.7% (95%CI 68.8–88.2) and Negative predictive value(NPV) 92.3% (95%CI 87.4–95.7) (Fig. 2). This implies that patients with a hematoma volume of 45.3 ml or less have an 80.82% sensitivity and 91.76% specificity for surviving with only medical treatment.

The previous meta-analysis suggests that there is no significant increase in the likelihood of death and dependency at 6 months for patients who undergo surgical treatment (OR 1.23; 95%CI 0.77–1.98) [23]. McKissock et al. indicate a tendency toward a greater risk of death and dependency following surgery (OR 1.20; 95%CI 0.83–1.74) [23]. Conversely, an alternative meta-analysis, which compiled data from the studies of Juvela et al. [24], Auer et al. [25], and Batjer et al. [26], demonstrates a trend indicating improved mortality (OR 0.50; 95%CI 0.28–0.92) [27] associated with surgical intervention. Surgery performed within 8 hours from onset appears to be beneficial [28]. In this study, it was observed that among patients undergoing surgical intervention with varying hematoma volumes, the surgical group with less than 30 ml exhibited significantly higher mortality than survival. Additionally, the 30-39.9 ml surgical group showed a trend towards higher mortality than survival, although the difference was not statistically significant. This suggests that surgical intervention in the group with volumes less than 40 ml may be associated with a significantly higher mortality rate. For the 40-49.9 ml group and the 50-59.9 ml group, there was a tendency suggesting a benefit for surgery in terms of survival over mortality, but these trends were not statistically significant. Despite variations in individual cerebral cortex volume and cranial cavity space, there may be additional factors influencing intracranial pressure within this volume range. Due to the limited population within this subset, further investigation could be warranted.

In the surgical group with hematoma volumes of 60 ml or more, there was a notable trend toward increased survival, which was statistically significant. Hematoma volumes of 60 ml or more could potentially increase ICP, which cannot be managed through autoregulation. For a cutoff of 60 ml, the indication for surgery improved the survival rate with a sensitivity of 80.36% (95% CI 67.6–89.8), a specificity of 72.46% (95% CI 60.4–82.5), a positive likelihood ratio of 2.92 (95% CI 1.95–4.37), a negative likelihood ratio of 0.27 (95% CI 0.16–0.47), a PPV of 70.3% (95% CI 57.6–81.1), and a NPV of 82.0% (95% CI 70.0-90.6). Notably, surgical intervention for hematoma volumes surpassing 60 ml also demonstrated a statistically significant benefit in saving lives in cases of basal ganglia hemorrhage.

**Fig. 3:**
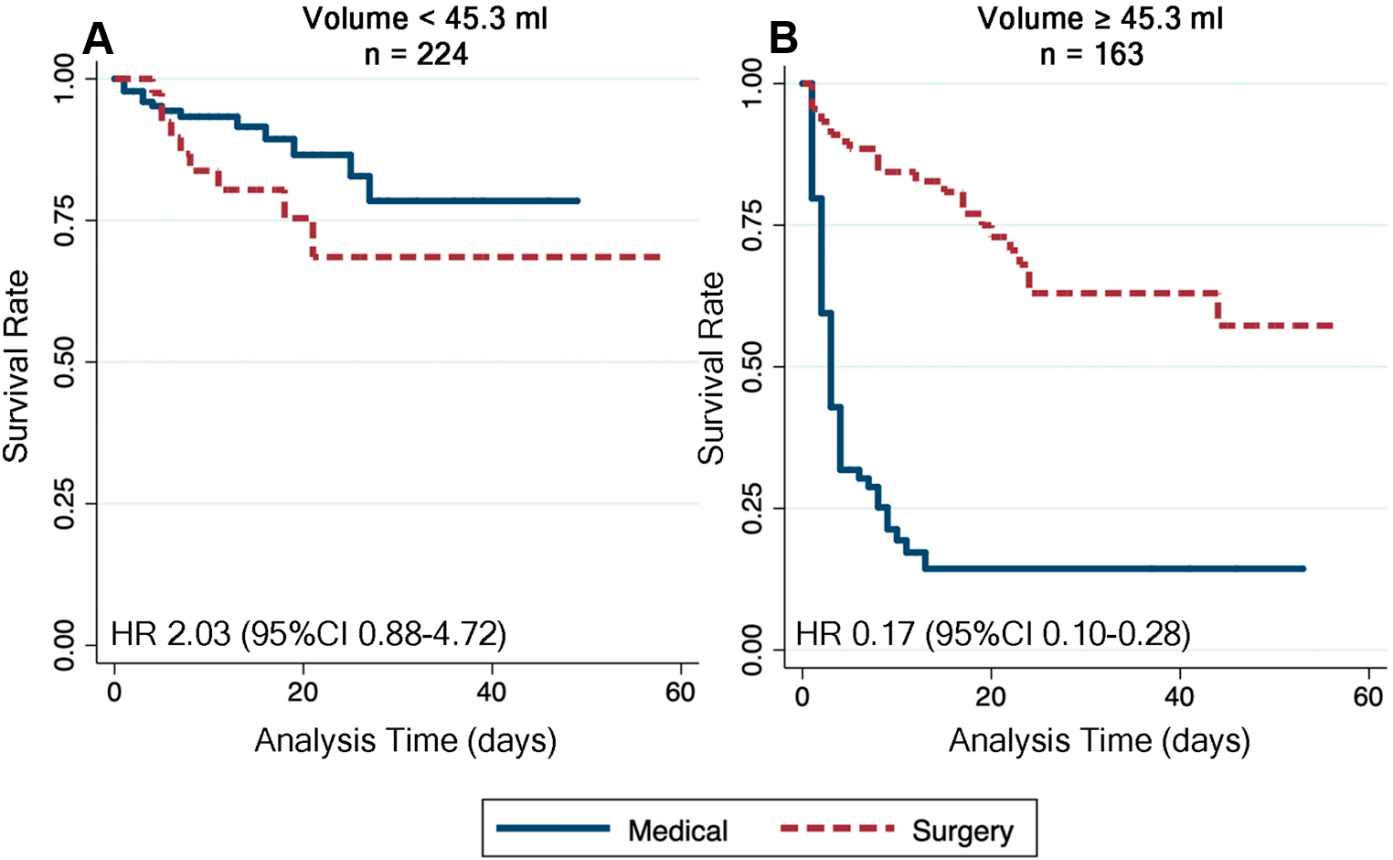
Kaplan–Meier survival analysis of 30-day survival following basal ganglia hemorrhage, stratified by treatment strategy. (**A**) Hematoma volume less than 45.3 mL group. (**B**) Hematoma volume at least 45.3 mL group

The Kaplan–Meier survival analysis comparing 30-day survival rates between patients with hematoma volumes less than 45.3 mL and those with volumes at least 45.3 mL revealed distinct differences based on treatment strategy. In the group with hematoma volumes less than 45.3 mL, surgical intervention was associated with an increased hazard ratio (2.03) and a borderline significant risk of mortality compared to medical treatment (*p* = 0.098). Conversely, in patients with hematoma volumes at least 45.3 mL, surgical intervention showed a clear benefit, with a reduced hazard ratio of 0.17 for mortality compared to medical treatment. Furthermore, for patients with hematoma volumes between 45.3 and 60 mL, additional factors such as GCS and pupil reactivity should be considered to determine the potential benefits of surgical intervention (Fig. 3).

In a large cohort of patients with SICH, multivariate analysis indicated that a LOS did not significantly correlate with discharge mRS after adjusting for the relevant covariates. The analysis found that worse discharge mRS were significantly associated with older age, greater initial hematoma volume, lower initial GCS, and the need for mechanical ventilation [29].The paper describes the mean LOS, which was found to be 9.72 days in 76 non-surgery patients and 24 days in 34 surgery patients [30].Our study reports a non-significant improvement in the length of hospital stay among patients receiving surgical and medical treatment (aOR 1.01; 95%CI 0.99–1.02) (Table 5). Relevant recent papers support and conclude that surgical management does not improve the LOS.

Complications from SICH reveal several routes of infection, with pneumonia being the most common disease associated with dependent patients, particularly those with low GCS and high mRS [31]. The increasing odds ratio for pneumonia in the surgery group is significant. This can be explained by several factors, including low scores on independent mRS assessments and a higher prevalence of mechanical ventilation support cases in the surgery group compared to the medical group, as outlined in our paper. Santosh B. Murthy et al. noted a gradual increase in infection rates from 18.7% in 2002–2003 to 24.1% in 2010–2011, with pneumonia being the predominant nosocomial infection at 15.4%, followed by UTI at 7.9% [32]. Our study revealed UTI as the second most prevalent infection, occurring in 7.83% of cases, with no significant disparity between the surgery and medical groups. Additionally, patients with ICH exhibit increased susceptibility to sepsis due to immunosuppression and dysbiosis of the intestinal microbiota [33]. Sepsis occurs in both the surgery and medical groups with barely any difference, and it is not statistically significant (Table 6).

Ruijun Ji et al. developed predictive factors for poor functional outcomes linked to mRS scores ranging from 3 to 6, observed one year after ICH within the derivation cohort. These predictive factors encompass age, admission NIHSS score, GCS, blood glucose levels, ICH location, hematoma volume, and intraventricular hemorrhage [34]. The study suggests that while surgical intervention is a critical option in specific clinical scenarios, medical treatment tends to result in better overall functional outcomes and shorter LOS for basal ganglia hemorrhage survivors. The higher rate of pneumonia in the surgical group may reflect the risks associated with invasive procedures and prolonged immobility. Additionally, the functional independence reflected in mRS scores highlights the potential benefits of less invasive management. These results emphasize the need for individualized treatment strategies that balance the benefits of surgery against its risks, focusing on improving functional outcomes and minimizing complications. (Table 6) Furthermore, functional outcomes following surgical or medical treatment are significantly influenced by the initial neurological condition, involvement of the dominant hemisphere, and hematoma size. Our study revealed that GCS and hematoma volume play critical roles in determining neurological outcomes. (Tables 2, 3 and 6)

Several papers were developed outlining the criteria for surgery in ICH [8, 18, 21, 35–38]. Key factors considered included the hematoma volume, GCS, and midline shift. Our observations revealed variations in how the GSC was interpreted by different individuals. The hematoma volume proved advantageous, facilitating earlier decision-making. Consequently, our study highlights hematoma volume as the primary indicator for surgery and conservative treatment, aligning with Abino Luzzi et al.‘s systematic reviews, which specify that the surgical volume threshold is at least 60 ml. (Table 7)

**Table 7:** Indications for surgery as reported in various journals

Authors	Intervention	Type review	Number	Year	Indication for surgery
Abino Luzzi et al.[8]	Craniotomy	Systematic review	19 articles	2019	Supratentorial Location >60 mL and GCS score 4–8
Hao Liu et al. [18]	Craniotomy	Retrospective cohort	310	2014	Volume ≥ 30 ml and ICH scores of 1 or 2
Parry-Jones AR et al.[35]	Craniotomy	Retrospective cohort	860	2019	Volume ≥30 mL or GCS score < 8
Lin F et al.[36]	Craniotomy	Prospective cohort	158	2022	Volume ≥ 20 mL and GCS drop ≥ 2
khallaf, M et al.[37]	Craniotomy	Prospective cohort	66	2019	signs of brain herniation, midline shift > 5 mm, Hydrocephalus
Wang, N. et al.[38]	Craniotomy	Retrospective cohort	54	2022	Midline shift > 5 mm or Ventricular compression
Jiang, L. et al.[39]	Endoscopic	Randomized control trial	91	2024	Neurodeficit and Volume > 20–30 ml
Our study	Craniotomy	Retrospective cohort	387	2024	Volume ≥ 60 ml, regardless of the GCS Volume 45.3–59.9 ml, depend on the patient’s status Volume < 30 ml, not recommended for surgery

GCS, Glasgow Coma Scale

Previous studies using machine learning methods, such as artificial intelligence (AI) and deep learning have successfully detected and reported the classification of intracranial hemorrhage [39–41]. Future AI technologies have the potential to facilitate early treatment or intervention, predict prognostic functional neurological outcomes and mortality rates of ICH. These helpful advancements can lead to cost reductions through optimal referrals, the elimination of unnecessary treatments, and prompt decision-making regarding initial management at healthcare facilities.

This study has limitations to consider. First, there is a limitation regarding the follow-up duration, as it extends beyond 30 days without the mRS in assessing long-term outcomes due to the referral healthcare system. Surgical intervention does not improve short-term functional outcomes, as its primary goal is to improve survival. However, the impact on long-term functional outcomes remains inconclusive. Collecting and analyzing data on long-term outcomes could provide valuable insights for future research. Second, the population included in the study experienced hematoma volume expansion, which was not addressed in this paper. This expansion was observed in 19 cases, comprising 4.68% of the total basal ganglia patient population. However, it’s important to note that the cutoff point for conservative treatment, with a high specificity of 91.76%, was not affected by this exclusion. Lastly, the statistical analysis may be impacted by the presence of elderly patients who exhibit a higher degree of brain atrophy compared to the general population. This factor could influence survival rates in cases of high-volume hematoma without surgical intervention.

## Conclusions

The medical treatment alone is feasible for hematoma volumes within the 0 to 45.3 mL. For hematoma volumes between 45.3 mL and 59.9 mL, the decision to pursue surgical intervention depends on the surgeon’s judgment and various contributing factors. Furthermore, hematoma volumes of at least 60 mL strongly indicate the need for surgical intervention in patients with basal ganglia hemorrhage. This is primarily associated with improved survival rates, while functional outcome remains unaffected, as it strictly depends on pre-treatment neurological status. The volumetric threshold is a practical clue for prompt management for both medical and surgical care plans and overall healthcare cost effectively.

## Data Availability

No datasets were generated or analysed during the current study.

## Abbreviations

AI: Artificial Intelligence
aOR: Adjusted odd ratios
CKD: Chronic kidney disease
CAD: Coronary heart disease
GSC: Glasgow Coma Scale
ICH: Intracerebral hemorrhage
ICP: Intracranial pressure
IQR: Interquartile range
LOS: Length of hospital stay
mRS: Modified Rankin scale
NPV: Negative predictive value
PPV: Positive predictive value
ROC: Receiver Operating Characteristic
SICH: Spontaneous intracerebral hemorrhage
uOR: Unadjusted odd ratios
UTI: Urinary tract infection
